# Citrate as Cost-Efficient NADPH Regenerating Agent

**DOI:** 10.3389/fbioe.2018.00196

**Published:** 2018-12-21

**Authors:** Reinhard Oeggl, Timo Neumann, Jochem Gätgens, Diego Romano, Stephan Noack, Dörte Rother

**Affiliations:** ^1^Forschungszentrum Jülich GmbH, IBG-1: Biotechnology, Jülich, Germany; ^2^Aachen Biology and Biotechnology, RWTH Aachen University, Aachen, Germany; ^3^Department of Food, Environmental and Nutritional Sciences, University of Milan, Milan, Italy

**Keywords:** citrate oxidation, oxidoreductase screening, nicotinamide cofactor, reduction equivalent regeneration, NADPH regeneration, cofactor regeneration

## Abstract

The economically efficient utilization of NAD(P)H-dependent enzymes requires the regeneration of consumed reduction equivalents. Classically, this is done by substrate supplementation, and if necessary by addition of one or more enzymes. The simplest method thereof is whole cell NADPH regeneration. In this context we now present an easy-to-apply whole cell cofactor regeneration approach, which can especially be used in screening applications. Simply by applying citrate to a buffer or directly using citrate/-phosphate buffer NADPH can be regenerated by native enzymes of the TCA cycle, practically present in all aerobic living organisms. Apart from viable-culturable cells, this regeneration approach can also be applied with lyophilized cells and even crude cell extracts. This is exemplarily shown for the synthesis of 1-phenylethanol from acetophenone with several oxidoreductases. The mechanism of NADPH regeneration by TCA cycle enzymes was further investigated by a transient isotopic labeling experiment feeding [1,5-^13^C]citrate. This revealed that the regeneration mechanism can further be optimized by genetic modification of two competing internal citrate metabolism pathways, the glyoxylate shunt, and the glutamate dehydrogenase.

## Introduction

β-Nicotinamide adenine dinucleotide phosphate (NADPH) is an obligatory frequent cofactor for oxidoreductases, an important enzyme class in a manifold of academically and industrially relevant reactions (Straathof et al., 2002; Burton, 2003; Xu, 2005; Goldberg et al., 2007; Zheng et al., 2017). Unfortunately, NADPH is consumed in stoichiometric ratios during these reactions. An external NADPH addition is economically unfeasible, because it is a metastable and expensive molecule (Faber, 2011; Wu et al., 2013). Hence, several biological *in vitro* methods for cofactor regeneration (including also NADH) have been developed by using: (I) whole cell biocatalysis, (II) enzyme-coupled, or (III) substrate-coupled cofactor regeneration (van der Donk and Zhao, 2003; Wu et al., 2013; Marpani et al., 2017).

In the substrate coupled cofactor regeneration the backward reaction of a single NAD(P)H-dependent reduction is utilized by oxidizing a second auxiliary substrate under NAD(P) consumption (Kara et al., 2014; Marpani et al., 2017). However, the utilization of the backwards reaction lowers the catalytic activity of the enzyme of interest for the target reaction. An enzyme-coupled method supplements an additional enzyme together with a suitable co-substrate to the reaction for NADPH regeneration (Liese and Villela Filho, 1999; Goldberg et al., 2007; Hall and Bommarius, 2011; Rauter et al., 2015). Advantageously, this method allows the enzyme of interest to operate at full catalytic activity. Yet, the supplementation of an additional enzyme also increases process costs and is less economically feasible (Tufvesson et al., 2013).

Whole cell NADPH regeneration utilizes intracellular enzymes of the host organism (Wichmann and Vasic-Racki, 2005). Thereby, whole cell regeneration intrinsically inherits the advantages of an enzyme coupled approach, which are here combined with an inexpensive enzyme production. While several NADPH regenerating reactions are available in *Escherichia coli*, commonly only those associated with the central carbon metabolism of *E. coli* are considered in *in vitro* whole cell NADPH regeneration approaches. These are in particular glucose-6-phosphate dehydrogenase, glucose dehydrogenase, and isocitrate dehydrogenase (IDH) (Wichmann and Vasic-Racki, 2005; Blank et al., 2010; Hummel and Gröger, 2014; Spaans et al., 2015). Though the first two enzymes are most often utilized, an IDH application would be highly interesting due to its central metabolic position and high affinity for NADPH (Schwaneberg et al., 2001).

As such IDH found already an application in *in vitro* screening methods of different NADPH-dependent cytochrome P450 variants (Schwaneberg et al., 2001). In short, the cultivated whole cells were placed in an *in-vitro* environment together with the particular substrate, isocitrate, NADP^+^, dimethoxy-sulfoxide (DMSO) and polymyxin B sulfate. Polymyxin B sulfate was added to permeabilize the cells, while DMSO is supplemented to increase substrate solubility. The intracellular isocitrate dehydrogenase (IDH) of the host *E. coli* was utilized to regenerate NADPH by adding the expensive specialty chemical isocitrate to the system (Schwaneberg et al., 2001). Isocitrate is converted in one physiological step to 2-oxoglutarate, reducing NADP^+^ to NADPH. Likewise, other screening approaches have been developed, but they all suffer either from cost intensive NADP^+^ supplementation or require further additives to avoid diffusion limitation over the cell membrane. The latter might also impair the targeted enzyme activity (Klibanov, 1997; Faber, 2011; Gerhards et al., 2012; Bornadel et al., 2016).

**Figure 1:**
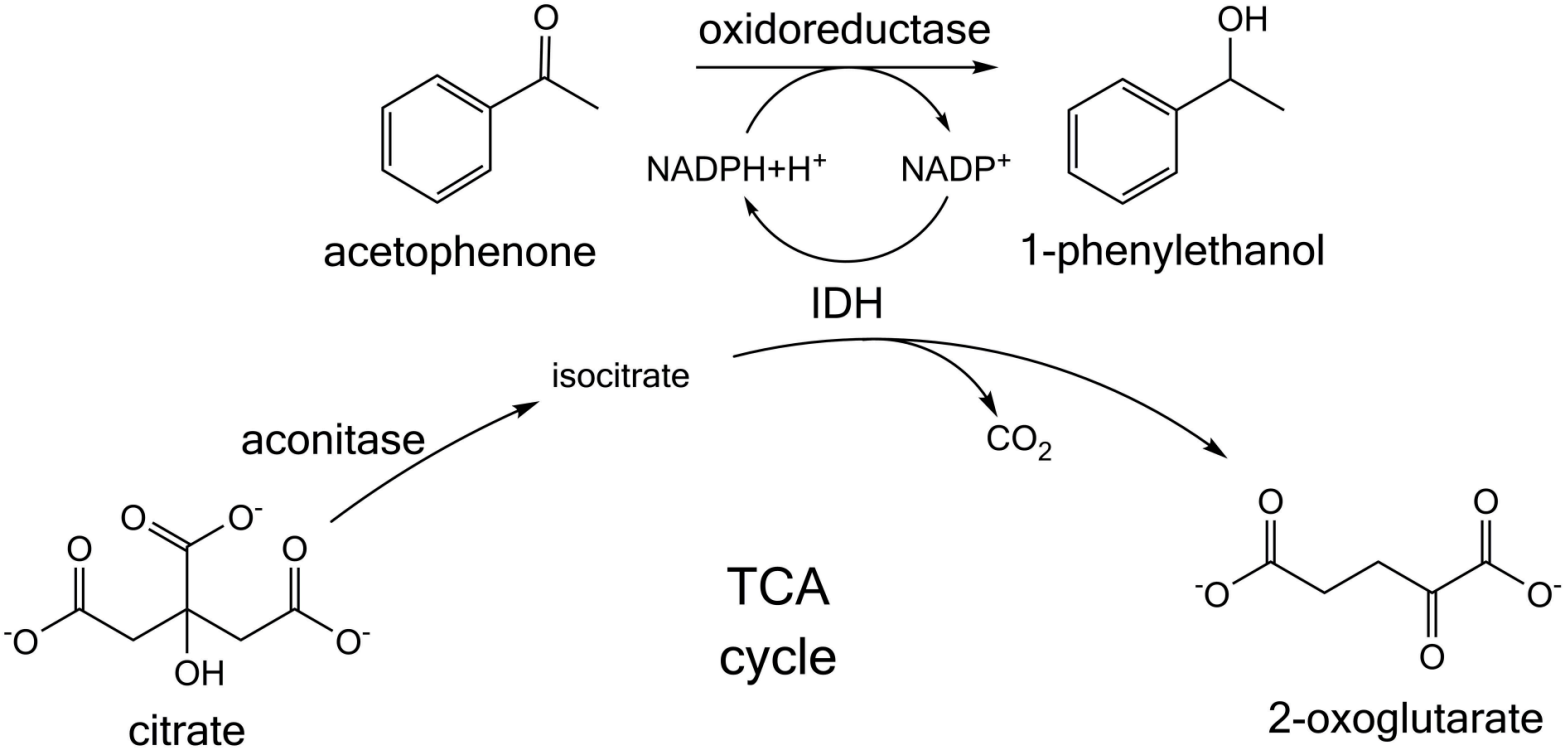
Citrate-dependent cofactor regeneration approach for screening NADPH-dependent oxidoreductases. Acetophenone is converted to 1-phenylethanol. In the reaction NADPH is consumed, which is proposed to be regenerated by the tricarboxylic acid (TCA) cycle enzymes present in whole cells or crude cell extract. Citrate is isomerized to isocitrate and then further converted by the enzyme isocitrate dehydrogenase (IDH). In this process IDH regenerates NADPH.

Building on this research, we present a whole cell NADPH regeneration in a targeted oxidoreductase reaction with the mundane bulk chemical citrate, which is converted by endogenous tricarboxylic acid (TCA) cycle enzymes. For this purpose, the specific activity of a target oxidoreductase reaction toward acetophenone was investigated in presence of citrate, when the enzyme is formulated as lyophilized whole cells (LWC) and crude cell extract (CCE) (Figure 1). Citrate is hypothesized to be isomerized by aconitase to isocitrate. Isocitrate is then decarboxylated by IDH to 2-oxoglutarate and thereby NADP^+^ is reduced to NADPH (Blank et al., 2010; Spaans et al., 2015). In this context it was investigated if native TCA enzyme levels are sufficient for a NADPH regeneration from citrate. Additionally, a transient isotopic labeling experiment with [1,5-^13^C]citrate was recorded to evaluate if citrate truly is converted along the proposed pathway and if competing side reactions occur. Citrate was also investigated as buffer substance with NADPH-regenerating activity to emphasize a possible application in NADPH-dependent enzyme screenings, without the need of further additives. To verify the global transferability of this proposed system, we investigated it with three different alcohol dehydrogenases, which were all heterologously introduced into *E. coli*. These were ketoreductase 1 from *Oogatea glycozyma* (KRED1-*Pglu*), alcohol dehydrogenase from *Ralstonia* sp. (RADH), and alcohol dehydrogenase from *Lactobacillus brevis* (*Lb*ADH).

## Materials and Methods

### Materials

All applied chemicals were purchased from Sigma Aldrich (Germany), if specified in purities ≥95%. The cell disruption by sonification was performed on a UP 200s ultrasonic processor (Hielscher Ultrasonics GmbH, Teltow, Germany) with an S1 sonotrode (Hielscher Ultrasonics GmbH, Teltow, Germany). The reactions were incubated in an Eppendorf thermomixer comfort (Eppendorf, Hamburg, Germany) and samples were centrifuged in an Eppendorf centrifuge 5424 (Eppendorf, Hamburg, Germany). Sample analysis was performed on an Agilent technologies 1260 infinity high performance liquid chromatography system (Agilent Technologies, Santa Clara, CA, USA). Metabolic profile analysis was performed on an Agilent Technologies 6890N gas chromatography system (Agilent Technologies, Santa Clara, CA, USA) coupled to a GCT Premier mass spectrometer (Waters Corporation, Milford, MA, USA). Pipetting assistance was provided by Tecan Freedom Evo 1 (Tecan, Männedorf, Swiss).

### Preparation of Biocatalysts

*Ogataea glucozyma* CBS 5766 ketoreductase 1 (KRED1-*Pglu*; GenBank: AKP95857.1) on a pET-26b(+) vector, *Ralstonia* species alcohol dehydrogenase (RADH; PDB: 4BMS_A) on a pET-21a vector and *Lactobacillus brevis* alcohol dehydrogenase (*Lb*ADH; GenBank: CAD66648.1) on a pET-22b vector were heterologously expressed in *E. coli* BL21 (DE3) (see Supplementary S1). All were cultivated in 1 L auto induction medium at 37°C and 90 rpm (Studier, 2005). After 4 h of cultivation, temperature was reduced to 20°C. Cells were harvested after 72 h by centrifugation at 7,000 g for 45 min at 4°C. Harvested cells were further processed to CCE. To prepare a CCE, the pellets were resuspended in 50 mM KP_i_ buffer pH 7.5, supplied with 0.1 mM MgCl_2_. Next, the cells were disrupted by sonication at an amplitude of 40 μm, and a cycle time of 0.5 s for 40 min at 4 to 8 °C. The solution was centrifuged at 8,000 g at 4°C for 45 min. The supernatant was transferred into a crystallization bowl and lyophilized at −54°C and 0.10 mbar. Lyophilized extract was mortared and stored at −20°C. The LWC catalyst was produced analogously, except cell lysis was not performed.

### Experimental Setup

All experiments below were conducted in a reaction volume of 1 mL. The activity of employed lyophilized whole cell (LWC) KRED1-*Pglu* was 0.1 U mg^−1^ and for the corresponding CCE 0.4 U mg^1^ in presence of 5 mM acetophenone and 10 mM citrate. *Lb*ADH LWC had an activity of 1.61 U mg^−1^ and as CCE 3.54 U mg^−1^. In case of RADH the LWC had an activity of 0.81 U mg^−1^ and for CCE 1.78 U mg^−1^. 1 U is defined 1 μmol acetophenone that is converted per min by the indicated enzyme amount at defined reaction conditions of 5 mM acetophenonen and 10 mM citrate.

#### Citrate Application in Whole Cell and Crude Cell Extract Setups

KRED1*-Pglu* reaction solutions contained 5 mM acetophenone, 0.1% (v/v) DMSO and 10 mM citrate suspended in 100 mM KP_i_ buffer pH 8. Six different conditions were tested: (I) 20 mg mL^−1^ LWC, (II) 20 mg mL^−1^ CCE, (III) 20 mg mL^−1^ LWC with 0.5 mM NADPH as first positive control, (IV) 20 mg mL^−1^ CCE with 0.5 mM NADPH as second positive control, (V) 20 mg mL^−1^ LWC as negative control without citrate addition, and (VI) 5 mg mL^−1^ purified enzyme. The whole setup was analogously performed with RADH at pH 8 and *Lb*ADH at pH 6.5. Reactions were incubated at 30°C and 1,000 rpm. Samples were prepared and analyzed as described below. The activity toward acetophenone was analyzed *via* HPLC. Experiments were conducted in triplets.

#### Supplementation of Isocitrate Dehydrogenase, Aconitase, and NADP^+^

A basic reaction solution was prepared containing 20 mg mL^−1^ KRED1*-Pglu* CCE in 100 mM KP_i_ buffer pH 8 with 5 mM acetophenone, 0.1% (v/v) DMSO, and 10 mM citrate. This basic solution was tested (i) alone, (ii) with 1 U mL^−1^ isocitrate dehydrogenase (IDH), (iii) with 1 U mL^−1^ aconitase, (iv) with both enzymes supplemented to the reaction solution, and (v) with 0.5 mM NADP^+^ supplemented. (vi) As negative control, the reaction solution was prepared without citrate and enzyme addition. The reaction was incubated at 30°C and 1,000 rpm. The screening was analogously performed for 20 mg mL^−1^ RADH under the same reaction conditions and 20 mg mL^−1^ *Lb*ADH with the KP_i_ buffer set to pH 6.5. All experiments in this section were produced in triplicates and pipetted with the aid of a robotic station. Samples were prepared and analyzed as described below. The activity toward acetophenone was analyzed *via* HPLC. Experiments were carried out in triplets.

#### [1,5-^13^C]Citrate Metabolic Profile Analysis

20 mg mL^−1^ KRED1-*Pglu* CCE were added to 40 mM KPi buffer pH 8 with 10 mM citrate acid-1,5-^13^C, 5 mM acetophenone, and 0.1% (v/v) DMSO. The reaction solution was incubated at 30°C and 1,000 rpm. Samples were taken after 0, 1, 2, 3, 4, and 5 h. A second reaction solution was prepared with 20 mg mL^−1^ *Lb*ADH CCE in 20 mM citrate acid-1,5-13C phosphate buffer pH 6.5 and 5 mM acetophenone (McIlvaine, 1921). Samples were taken after 0, 10, 20, and 30 min. A negative control of the *Lb*ADH reaction was prepared, applying the same conditions, without citrate addition. Samples were taken after 0 and 30 min. All samples were centrifuged at 20,000 g and 130 μL of the supernatant were transferred into a 1.5 mL reaction vessel, frozen in liquid nitrogen and then stored at −20°C. The samples were further processed for GC-MS metabolic profile analysis as described below. Measurements were performed in singlets.

#### Citrate-Phosphate Buffer Investigation

Citrate phosphate buffer was prepared by combining defined amounts of 200 mM K_2_HPO_4_ with 100 mM citrate solution. The pH of CP_i_ is adjusted by the ratio of the two solutions to each other (McIlvaine, 1921). 20 mg mL^−1^ KRED1-*Pglu* CCE was added to buffered solutions that all contained 5 mM acetophenone and 0.1% (v/v) DMSO, but differed in source and concentration as follows: (i) 20 mM citrate phosphate buffer pH 8, (ii) 100 mM citrate phosphate buffer pH 8, and (iii) 100 mM KP_i_ buffer pH 8 containing 10 mM citrate. The reaction solutions were incubated at 30°C and 1,000 rpm. The experimental setup was repeated analogously with RADH at pH 8 and *Lb*ADH at pH 6.5. The activity toward acetophenone was analyzed *via* HPLC. Measurements were performed in triplets.

### Analytics

Quantitative analysis was performed to determine initial activity rates in the range of maximum 10% of substrate conversion toward acetophenone on an HPLC. In case of *Lb*ADH, samples were taken after 0, 5, 10, 20, 30, and 40 min. In case of RADH and KRED1-*Pglu*, samples were taken after 0, 30, 60, 120, 150, and 180 min. Samples were diluted 1:25 in acetonitrile containing 100 μL L^−1^ toluene as internal standard and subsequently analyzed with a ChiralPak ID column (5 μm, 4.6 × 250 mm) at a flow of 1.0 mL min^−1^ under isocratic conditions of 50% acetonitrile and 50% water (purified by reverse osmosis, MilliQ) at 20°C. Retention times: 1-phenylethanol: 3.3 min (215 nm); acetophenone: 5.4 min (250 nm); toluene: 6.3 min (215 nm).

Qualitative untargeted GC-MS metabolic profile analysis was carried out under the same conditions as described by Paczia et al. (2012).

## Results and Discussion

### Citrate as Effective Agent for NADPH Regeneration in Oxidoreductase Reactions

In aerobic organisms citrate is oxidized in the TCA cycle, while regenerating redox-equivalents. In this study, we investigated if this pathway can also be accessed *in vitro* in LWC to provide NADPH for a targeted oxidoreductase reaction. Since citrate uptake in *E. coli* is administered by a membrane transporter CitT, also crude cell extract (CCE) was investigated to rule out a substrate transport limitation (Pos et al., 1998). To ensure an independency of obtained results from a studied alcohol dehydrogenase, the experimental setup was investigated with KRED1-*Pglu, Lb*ADH, and RADH individually. As model reaction for all three oxidoreductases served the reduction of acetophenone to 1-phenylethanol under NADPH consumption (herein called target reaction; Figure 1; Leuchs and Greiner, 2011; Kulig et al., 2013; Contente et al., 2015, 2016). If citrate is converted by endogenous TCA cycle enzymes NADPH would be generated and thus a 1-phenylethanol formation would be observable. The possibility of endogenous NADPH levels to suffice for catalytic activity or other endogenous sources to regenerate NADPH was investigated by a LWC setup with acetophenone, but without citrate addition. The possibility that citrate could also be converted by the applied alcohol dehydrogenases was ruled out by performing the model reaction with purified KRED1-*Pglu, Lb*ADH, and RADH individually. Also NADPH was supplemented instead of citrate to rule out a limitation of available NADPH.

**Figure 2:**
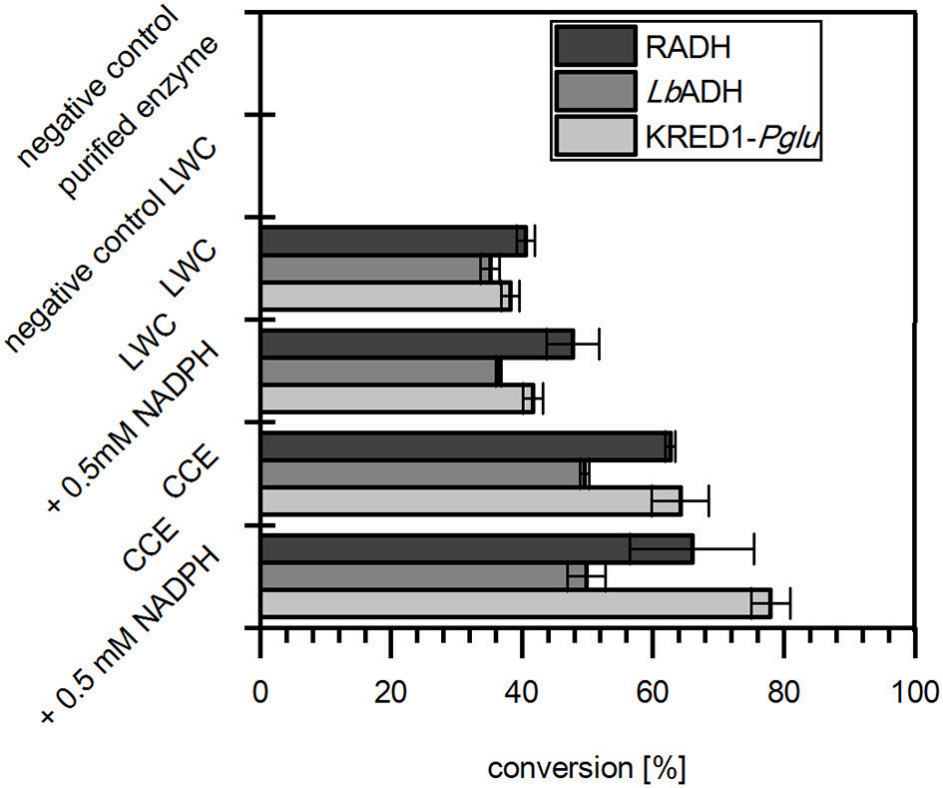
Citrate operates as redox-equivalent regenerating agent in product synthesis. The capability of citrate to regenerate NADPH in a targeted oxidoreductase reaction was investigated with *Ogataea glucozyma* CBS 5766 ketoreductase1 (KRED1*-Pglu*), *Lactobacillus brevis* alcohol dehydrogenase (*Lb*ADH), and *Ralstonia* sp. alcohol dehydrogenase (RADH) as lyophilized whole cell (LWC) and crude cell extract (CCE) catalysts. Therefore, citrate was provided to regenerate through endogenous tricarboxylic acid cycle enzyme NADPH, which is consumed by the targeted oxidoreductases in the reduction of acetophenone. Only the “negative control purified enzyme” contained the oxidoreductases in purified form instead of LWC/CCE. The “negative control LWC”: contained all compounds except citrate. The reaction setups with “0.5 mM NADPH” contained NADPH instead of citrate. *n* = 3.

In the results all three targeted oxidoreductases were active in LWC and CCE formulation when citrate was present and reduced acetophenone (Figure 2). In absence of citrate LWCs did not convert any acetophenone, which proves that citrate is neither substrate of the selected oxidoreductases nor does citrate directly reduce NADP^+^. Moreover, the reaction with purified enzyme also did not display any activity, which demonstrates that citrate is not converted by the target oxidoreductase to gain NADPH. Together this indicates strongly, that citrate is converted by endogenous TCA cycle enzymes of both the CCE and LWC. More importantly, it verifies that citrate is applicable *in vitro* as inexpensive cosubstrate to achieve good product conversion by whole cell NADPH regeneration.

**Figure 3:**
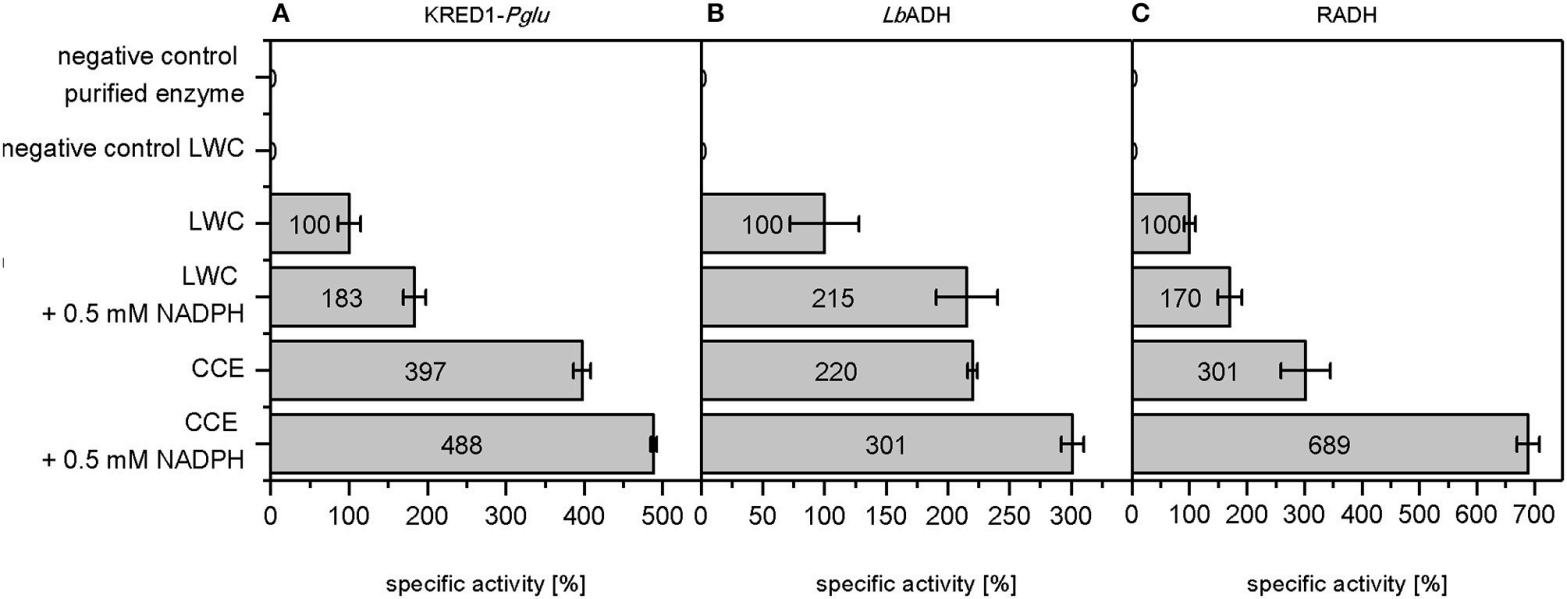
Relative initial activities gained by whole cell regeneration from citrate. The capability of citrate to regenerate NADPH in a targeted oxidoreductase reaction was investigated with **(A)** *Ogataea glucozyma* CBS 5766 ketoreductase1 (KRED1*-Pglu*), **(B)** *Lactobacillus brevis* alcohol dehydrogenase (*Lb*ADH), and **(C)** *Ralstonia* sp. alcohol dehydrogenase (RADH) as lyophilized whole cell (LWC) and crude cell extract catalysts. The “negative control purified enzyme” contained the oxidoreductases in purified form instead of LWC/CCE. The “negative control LWC”: contained all compounds except citrate. The reaction setups with “0.5 mM NADPH” contained NADPH instead of citrate. 0.10 U mg^−1^ KRED1-*Pglu* LWC, 1.61 U mg^−1^ *Lb*ADH LWC, and 0.81 U mg^−1^ RADH LWC were normalized to 100% specific activity. *n* = 3.

A more detailed look at relative initial activities under the investigated conditions demonstrates CCEs to be more active than LWCs (Figure 3). This indicates indeed mass transport of citrate as limiting to some extent. A prior cell lysis to synthesis application may thus circumvent such a limitation and make an application of CCE recommendable.

An additional observation shows higher initial activities by direct NADPH supplementation than with CCE (Figure 3). This indicates that under citrate addition the reaction is limited in K_M_ for NADPH. For this matter three factors are conceivable: (i) endogenous NADP^+^ amounts are too low, (ii) the present TCA cycle enzymes do not suffice to regenerate NADPH fast enough, and (iii) untargeted side-reactions in the CCE and LWC setup may compete with the targeted oxidoreductase reaction for NADPH. In all three cases an excess of NADPH saturates the demand of all NADPH-dependent enzymes in the reaction, allowing the targeted oxidoreductases to operate at V_max_.

In conclusion these first findings demonstrate citrate as a well applicable, inexpensive cosubstrate for NADPH regeneration during product synthesis. A detailed analysis of initial activities shows that a mass transport limitation for citrate reduces activities in LWCs. Hence, an application of this NADPH regeneration method would be preferred in CCE. During initial activity analysis it was also observed that all investigated enzymes do not operate at V_max_, which was revealed by the addition of 0.5 mM NADPH. Subsequently, the reason for this limitation is investigated on a cellular basis. Here, three different circumstances were proposed as limiting an operation at V_max_: (i) too low endogenous NADP^+^ levels, (ii) too low TCA cycle enzyme expression, or (iii) untargeted side-reactions that deplete NADPH.

### TCA Cycle Enzymes and Endogenous NADP^+^ Levels Are Sufficient for NADPH Regeneration

**Figure 4:**
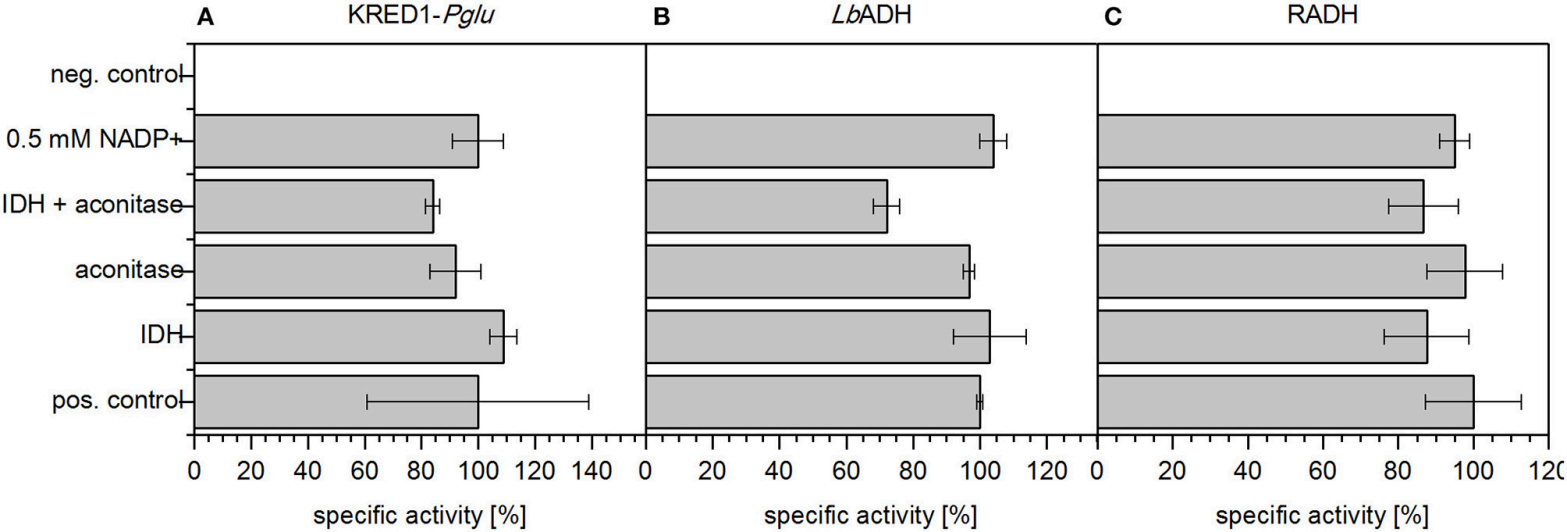
Limitation analysis based on endogenous NADP^+^ amounts and endogenous TCA cycle enzymes. In an applied CCE setup the reduction of acetophenone by **(A)** *Ogataea glucozyma* CBS 5766 ketoreductase 1 (KRED1-*Pglu*), **(B)** *Lactobacillus brevis* alcohol dehydrogenase (*Lb*ADH), and **(C)** *Ralstonia* sp. alcohol dehydrogenase (RADH) was investigated in presence of citrate. To identify a possible limitation by endogenous TCA cycle enzymes 1 U mL^−1^ aconitase and 1 U mL^−1^ isocitrate dehydrogenase (IDH) were added individually and together. Further also the possibility of limiting NADP^+^ amounts was investigated by supplementation thereof. The negative control (neg. control) contained neither additional enzymes for cofactor regeneration nor citrate; 0.34 U mg^−1^ KRED1-*Pglu* CCE, 3.56 U mg^−1^ *Lb*ADH CCE, and 2.4 U mg^−1^ RADH CCE were normalized to 100% specific activity. *n* = 3.

First the potential of an inherent limitation caused by either low basal levels of the involved TCA cycle enzymes aconitase and IDH (Figure 1) or insufficient endogenous NADP^+^ levels was investigated. To investigate the first case, both enzymes were supplemented in CCE experiments individually as well as in combination (Figure 4). For the second case 0.5 mM NADP^+^ were supplemented to a CCE setup. The negative control neither contained citrate nor any supplemented aconitase, IDH, or NADP^+^. As positive control the CCE of each of the three oxidoreductases KRED1-*Pglu, Lb*ADH, and RADH, was added to a buffered reaction solution which contained acetophenone and citrate, but no additional enzymes.

The recorded initial activities of the positive control and supplemented aconitase and IDH both individually and combined were approximately equally fast (Figure 4). This indicates endogenous enzyme levels as indeed sufficient for citrate metabolism. Literature supports this claim as IDH is naturally well overexpressed in *E. coli* (Vasquez and Reeves, 1979; Schwaneberg et al., 2001).

The supplementation of NADP^+^ also did not affect any activity increase (Figure 4). This indicates NADP^+^ also as not limiting in the setup. The finding is reinforced by an estimation of endogenous NADP^+^ amounts based on literature values published by Bennett et al. (2009) and Chemler et al. (2010). Based on their values NADP^+^/NADPH molecules were estimated to be in excess by a magnitude of 10^7^ to the targeted oxidoreduteases (estimation see Supplementary S3.1).

Together these findings rule out a possibility of a limitation caused by insufficient TCA enzymes to regenerate NADPH. Further, intrinsically provided NADP^+^ levels are abundant enough as to not limit catalytic rates of the targeted oxidoreductase reactions. Thus, the potential of undesired side reaction as cause for the observed limitation has to be investigated.

### [1,5-^13^C]Citrate Metabolic Profile Analysis Identifies Limiting Side Reactions

To identify if citrate is funneled into side reactions a transient isotopic labeling experiment with ^13^C-labeled citrate was performed. Acetophenone and [1,5-^13^C]citrate were added to a buffered reaction mixture with *Lb*ADH CCE catalyst (for the same experiment performed with KRED1-*Pglu* see Supplementary S2.3). A GC-ToF-MS analysis recorded the consumption of citrate, the formation of 1-phenylethanol and, most importantly, for the specific labeling enrichment in several TCA cycle intermediates downstream of citrate. Any further implications from citrate conversion, which did not directly affect NADPH regeneration, are beyond the scope of this study and were thus not considered.

**Figure 5:**
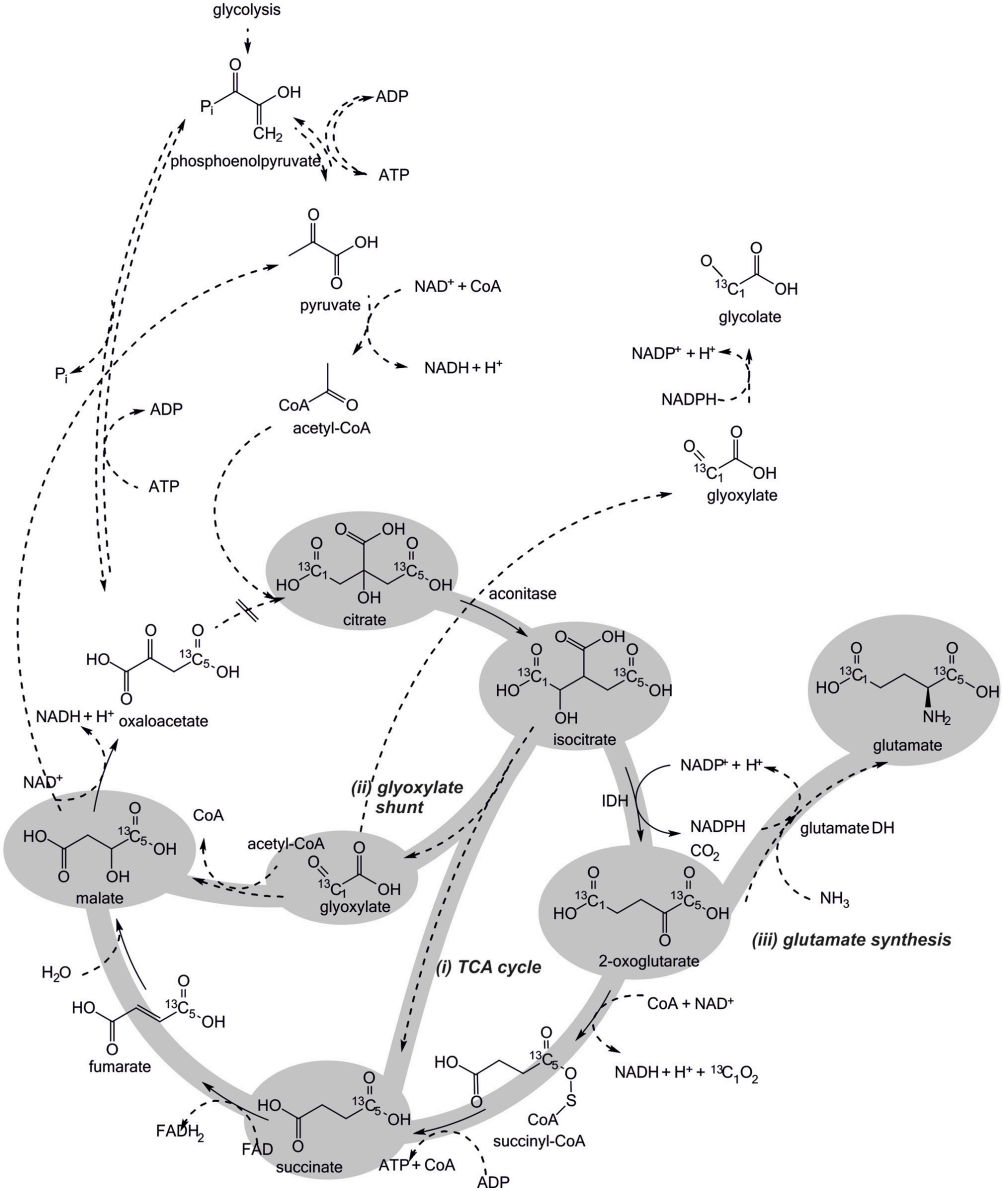
Potential routes of citrate metabolism in *Escherichia coli* BL21 crude cell extract. The ^13^C-labeling introduced via citrate is distributed among the depicted intermediates, depending on which enzymes are active under CCE conditions. All four routes contribute differently to NADPH regeneration. The first step of the TCA cycle, the condensation of oxaloacetate and acetyl-CoA to citrate is presumably not active in CCE.

In principle, three proximate competing routes to the conversion of citrate along the TCA cycle are conceivable (Figure 5): (i) 2-oxoglutarate is partly reduced to glutamate by glutamate dehydrogenase (GDH); (ii) isocitrate is partly split into succinate and glyoxylate, which both are further converted to malate and (iii) glyoxylate is partly reduced to glycolate by glyoxylate reductase (GR; Ornston and Ornston, 1969; Csonka and Fraenkel, 1976; Spaans et al., 2015).

Ideally, only the TCA cycle is involved in citrate metabolization, because only by this mode NADPH is regenerated in the targeted manner by IDH. Accordingly, ^13^C-labeling would be introduced into the TCA cycle intermediates and malate would accumulate. In the first competing case, the GDH reaction would partly consume the NADPH formed by IDH, resulting in an overall lowered NADPH regeneration capacity and an accumulation of glutamate. In the second case, NADPH regeneration by IDH would be partly skipped and, again, accumulation of malate is expected. Finally, the third case would be the worst one, since the GR reaction would directly compete for the available NADPH pool without adding any reduction equivalents to it (Figure 5).

**Figure 6:**
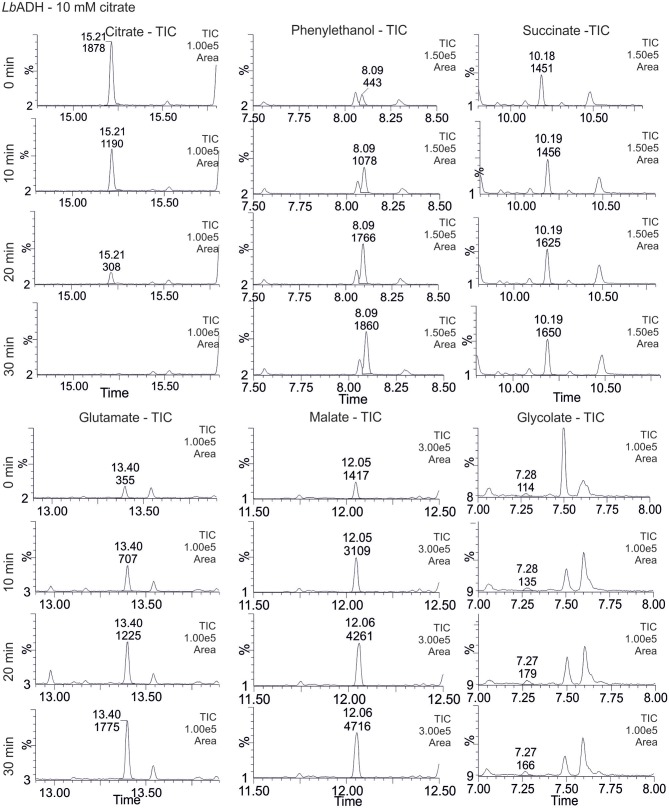
Total ion chromatograms (TIC) of selected TCA cycle intermediates. Relative concentration changes during the isotopic labeling experiment with [1,5-^13^C]citrate in *Lactobacillus brevis* alcohol dehydrogenase (*Lb*ADH) crude cell extract are shown (for corresponding mass spectrometic chromatogram of all detected metabolites see Supplementary S2.1 and for corresponding results of KRED1-*Pglu* see Supplementary S2.3).

From the total ion chromatograms (TIC) it can be seen that citrate is completely converted within the first 20 min of the reaction (Figure 6). In the same pattern the product concentration of the target reaction, 1-phenylethanol, increases. The TCA cycle intermediate succinate was already present at the start of the reaction and no significant change was observed. By contrast, malate and glutamate strongly accumulated along the CCE experiment. For glycolate, only a minor elevation was recorded over time (Figure 6). The corresponding negative control, which contained all reactants like the reaction solution except for citrate, did not exhibit any product formation and no significant elevation of TCA cycle intermediates (Supplementary S2.2).

The obtained TIC findings suggest all presented pathways for citrate metabolism (Figure 5) to be indeed active under CCE conditions. Foremost, the observation of 1-phenylethanol in the reaction solution (Figure 6) and its absence in the negative control (Supplementary S2.2) substantiates the theory of an active TCA cycle, because required NADPH can only be gained by a conversion through IDH. The accumulation of glutamate and glycolate also indicates a presence of side-reactions caused by GR and GDH, which lower NADPH amounts for the target oxidoreductase reaction. To validate an occurrence of these side reactions the transition of the ^13^C labeled carbon atom in the metabolite profile was investigated.

**Figure 7:**
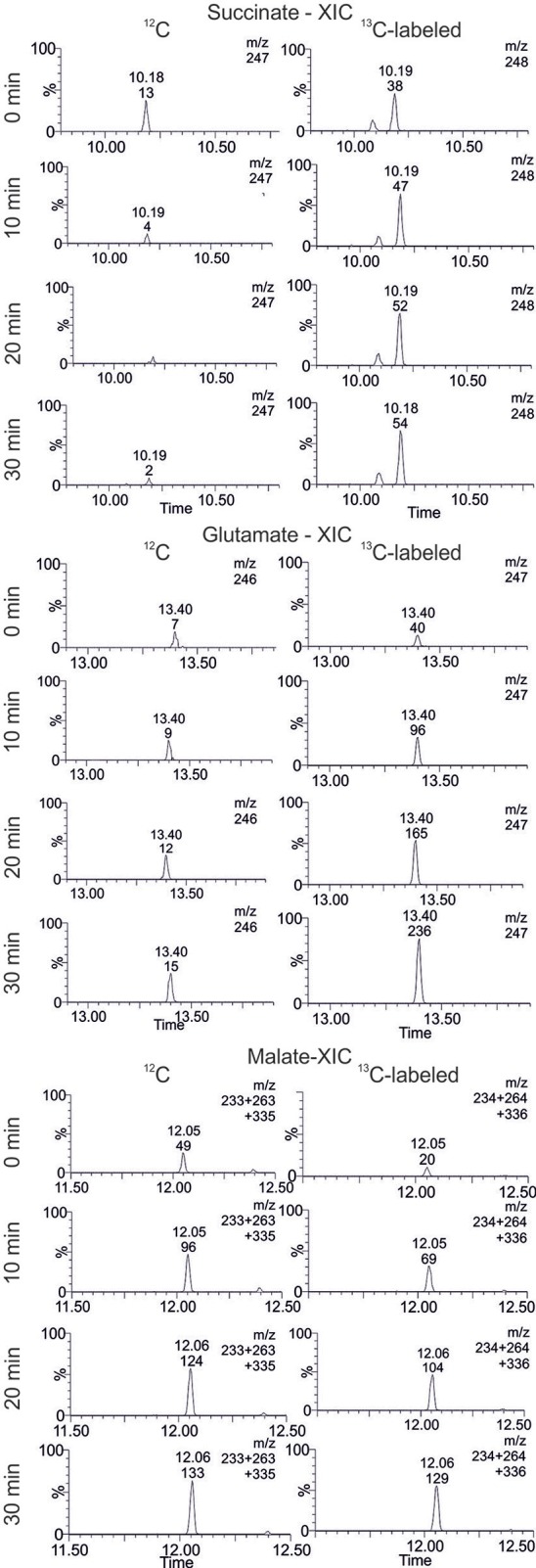
Extracted ion chromatogram (XIC) of selected TCA cycle intermediates. The accumulation and dissipation of ^12^C and ^13^C labeled species during the isotopic labeling experiment with [1,5-^13^C]citrate in *Lactobacillus brevis* alcohol dehydrogenase (*Lb*ADH) crude cell extract are displayed (corresponding results of KRED1-*Pglu* see Supplementary S2.4).

In extracted ion chromatograms (XIC) the distinct mass fragments for the unlabeled and ^13^C labeled metabolites were tracked. Firstly, unlabeled succinate dissipates over the reaction time, while ^13^C-labeling from citrate is introduced *via* the TCA cycle and glyoxylate shunt (Figure 7). This indicates a continuous turnover of the succinate pool, leading to a stable metabolite level during the CCE experiment (Figure 5). For malate and glutamate, the fractions of ^13^C-labeled and unlabeled species elevated to an equal extent, indicating that both metabolites represent dead-end pools for (i) citrate metabolism along the TCA cycle, (ii) the GDH by-pass, and (iii) the glyoxylate shunt. Thus, the selected XIC verifies all three citrate pathway as active. Yet, it did not grant quantitative information on the distribution ratio of citrate metabolism between the three pathways. An estimation of the extent of the GDH by-pass and the glyoxylate shunt is nonetheless possible with literature values. In the gloxylate shunt acetyl-CoA is required to obtain malate (Figure 5). Based on literature values the acetyl-CoA concentration in the setup can be estimated to range of 0.04 to 0.03 mM (estimation Supplementary S3.2; Bennett et al., 2009). Accordingly, only 0.4% of citrate would dissipate in this pathway. This estimation adjoined with the insignificant findings of GDH product in the TIC chromatogram indicates the glyoxylate shunt as dissipation source for citrate as negligible. In case of glutamate such an estimation is not possible and any ratio on the loss would be speculative. Yet, *E. coli* is a strong glutamate producer, which is indicated by the fact that the most abundant intracellular *E. coli* metabolite is glutamate (Bennett et al., 2009). A loss in this side reaction could be substantial in dependence of available NH_3_, which is unfortunately unknown. The ratio of this side reactions could be clarified in the future with a [2,4-^13^C]citrate tracer, which gives a distinct ^13^C-labeling pattern for the origin of each metabolite (see Supplementary S4).

In summary, the transient ^13^C isotope labeling verified the theory of NADPH regeneration through TCA cycle enzymes. However, the citrate metabolism into the glyoxylate shunt, glycolate synthesis, and glutamate synthesis were identified as competing side reactions (Figure 5). The glyoxylate shunt and glycolate synthesis was determined of minor to insignificant importance in the citrate depletion, because required intracellular acetyl-CoA levels are estimated to be too low to substantially promote a conversion of citrate through this pathway. The extent of NADPH loss by glutamate could unfortunately not be quantified, as the concentration of ammonia in the setup was unknown. Yet, all herein obtained findings indicate glutamate synthesis as limitation cause for NADPH regeneration efficiency. Interestingly, by the sheer quantity of present targeted oxidoreductase to all other host enzymes it could be assume to be more likely that the majority of NADPH is consumed by the target oxidoreductase. Acetophenone, however, is an artificial substrate for the targeted oxidoreductases (KRED1-*Pglu, Lb*ADH, RADH), while citrate and the metabolites of the TCA cycle are natural substrates of the primary metabolism. Thus, it can be speculated that the host enzymes and in this particular case the GDH exhibit a higher NADPH turnover rate than the targeted oxidoreductase. In future applications this issue could be solved by genetically engineering the efficiency of the glutamate synthesis or introducing an ammonium limitation that would slow GDH synthesis and thereby the depletion of NADPH. Also the inactivation of the glyoxylate shunt in *E. coli* by cultivating it on glucose as sole carbon and energy source could enhance the NADPH regeneration efficiency from citrate (Noronha et al., 2000; Phue and Shiloach, 2004; Phue et al., 2005).

In summary these gained findings illustrate very well that citrate is capable to provide NADPH to target product conversions. An application in investigation of initial activity rates is presently limited by the reported side reactions. However, a relative initial activity comparison may already be applicable, and implementation of necessary genetic alterations could unfold the full potential of this method.

### NADPH Regenerating Buffer for Initial Activity Measurements of Oxidoreductases

The potential for initial activity comparison by this method could be increased by applying citrate as *in vitro* reaction environment in form of citrate-phosphate (CP_i_) buffer. This buffer is a universal buffer, which covers a pH range from 2 to 8. Interestingly, this it would allow a relative activity comparison of oxidoreductases in screenings without the need to switch buffer types. Especially, because buffer types are reported to also affect oxidoreductase activity (Zhao, 2005; Kulig et al., 2013). All three targeted oxidoreductases were applied as CCE in 100 mM CP_i_ buffer and 20 mM CP_i_ buffer and compared against 100 mM KP_i_ buffer with 10 mM citrate supplementation.

**Figure 8:**
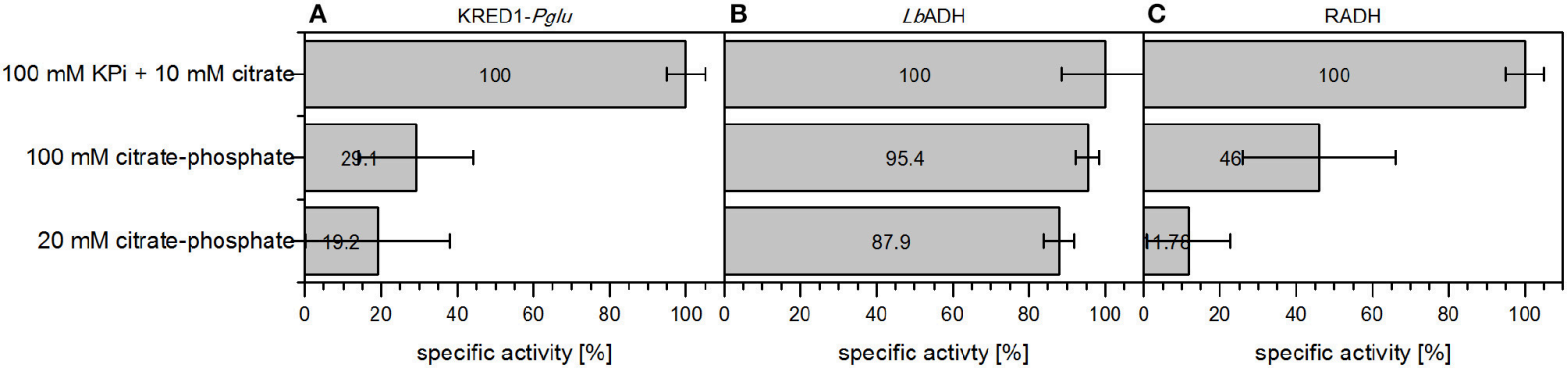
Comparison of citrate-phosphate buffer against citrate addition in terms of specific initial activities of different oxidoreductases. The required cofactor for the NADPH-dependent oxidoreductases is regenerated by either 10 mM citrate supplementation in an environment using 100 mM KP_i_ buffer or directly applying citrate as 20 or 100 mM citrate-phosphate buffer. **(A)** was performed with KRED1-*Pglu* at pH 8, **(B)** was performed with *Lb*ADH at pH 6.5, and **(C)** was performed with RADH at pH 8. 0.34 U mg^−1^ KRED1-*Pglu* CCE, 3.56 U mg^−1^ *Lb*ADH CCE, and 2.4 U mg^−1^ RADH CCE were normalized to 100 % specific activity. *n* = 3.

KRED1-*Pglu* and RADH CCE exhibited only reduced specific activity in 100 mM CP_i_ and accordingly with 20 mM CP_i_, when compared to the standard reaction conditions (Figures 8A,C). For *Lb*ADH almost no activity loss was observed, when 100 mM CP_i_ was compared against the standard conditions (Figure 8B). Even at 20 mM CP_i_ only a minor decline in activity was determined.

This observation could be caused by the pH-dependent buffer composition of the reactions. Both KRED1-*Pglu* and RADH have their pH optimum for reduction at 8, while *Lb*ADH performs best at pH 6.5 (Leuchs and Greiner, 2011; Kulig et al., 2013; Contente et al., 2015). CP_i_ buffer consists of a distinct mixture of a K_2_HPO_4_ and a citrate stock solution. The acidic citrate part represent only a minor fraction at a higher pH (McIlvaine, 1921). Hence, 100 mM CP_i_ pH 8 contains 1.2 mM citrate and 20 mM CP_i_ only 0.24 mM. At pH 6 citrate is present in 13 mM concentrations in 100 mM CP_i_ buffer and in the 20 mM setup 2.6 mM concentrations. The results for *Lb*ADH indicate this concentration of 2.6 mM in the 20 mM CP_i_ setup as sufficient for a catalytic comparison. This concentration is calculated to also be present in 100 mM CP_i_ buffer pH 7.7 (McIlvaine, 1921). Hence, 100 mM CP_i_ buffer would allow a pH screening under consistent buffer components in a range of pH 2 to 7.7. The clear advantage for screenings is, that effects of changing buffer salts can be neglected (Zhao, 2005; Kulig et al., 2013).

Conclusively, CP_i_ offers the possibility of a universal buffer environment for oxidoredcutase applications in a buffer range of pH 2 to 7.7. This could for instance presently be employed for relative activity comparisons. However, it should be treated cautiously as the applied enzymes in this study exhibit different activities toward the used artificial substrate acetophenone. Thus, the regeneration capacity for NADPH in the present stage may be insufficient for reactions with high V_max_.

## Conclusion

In summary, the cheap bulk chemical citrate was proven to regenerate NADPH by conversion through the endogenous TCA cycle in a CCE and LWC setup. CCE setups yielded higher specific activities than LWCs, most likely due to the absence of a diffusion barrier. An analysis of initial activities revealed a limitation of the recombinant oxidoreductases, which was thought to be due to too low NADPH levels to achieve V_max_. Here, endogenous TCA enzyme levels and NADP^+^ amounts could be excluded as limitation origin as experiments found them to be present in abundance. A transient isotopic labeling experiment feeding [1,5-^13^C]citrate verified citrate dissipation in the glyoxylate shunt to a minor extent and a supposedly significant loss of NADPH in glutamate synthesis. In the future, the glyoxylate shunt could be inactivated through cultivation of *E. coli* on glucose as sole carbon source. Glutamate synthesis could be lowered by feeding only defined amounts of ammonia. Apart from the characterization of the citrate regeneration, citrate was also successfully applied as CP_i_ reaction buffer for targeted oxidoreductase reactions. Hence, citrate is proposed as applicable in comparative activity screenings of oxidoreductases in a range of pH 6.5 to 7.7. Advantageously, this allows an inexpensive NADPH regeneration in a simple reaction setup, without the need of extensive additives. This may become even more appealing once necessary genetic optimizations in *E. coli* have been made. Nonetheless, the described method is already in the present form well applicable to produce satisfying conversions in product synthesis. Notably, this study also uncovered that further steps of the TCA cycle are active, in which also NADH is regenerated. Though this was not further investigated in this study, the application of citrate may even be broadened to NADH-dependent enzymes.

## Author Contributions

RO and DöR designed the study and prepared the manuscript. The enzyme KRED1-*Pglu* from *Ogataea glucozyma* CBD 5766 was contributed by DiR. TN carried out the experimental setup and procedure. RO, TN, and JG carried out the analytics and evaluation of results. RO and DöR supervised the experimental work. RO, JG, DiR, SN, and DöR helped to finalize the manuscript. All authors approved the final manuscript.

### Conflict of Interest Statement

The authors declare that the research was conducted in the absence of any commercial or financial relationships that could be construed as a potential conflict of interest.

## Abbreviations

CCE: crude cell extract
DMSO: dimethyl-sulfoxide
GDH: glutamate dehydrogenase
GR: glyoxylate reductase
*Lb*ADH: *Lactobacillus brevis* alcohol dehydrogenase
LWC: lyophilized whole cells
IDH: isocitrate dehydrogenase
NADH, β-1: 2-nicotinamide adenine dinucleotide
NADPH, β-1: 2-nicotinamide adenine dinucleotide phosphate
KRED1-*Pglu*: *Ogataea glucozyma* CBS 5766 ketoreductase 1
RADH: *Ralstonia* species alcohol dehydrogenase
TCA: tricarboxylic acid
TIC: total ion chromatogram
XIC: extracted ion chromatogram.
