# Supplementary material for: Citrate as Cost-Efficient NADPH Regenerating Agent
Source: Front Bioeng Biotechnol. 2018 Dec 21;6:196. doi: 10.3389/fbioe.2018.00196 (PMC6315136; doi:10.3389/fbioe.2018.00196)
Supplement: Supplementary file 1 [file Presentation_1.pdf]

Supplementary Material

Citrate as cost-efficient NADPH regenerating agent

Reinhard Oeggl[1], Timo Neumann[1], Jochem Gätgens[1], Diego Romano[2], Stephan Noack[1], Dörte Rother[1,3*]

[1] Forschungszentrum Jülich GmbH, IBG-1: Biotechnology, Wilhelm-Johnen Straße, 52425 Jülich, Germany

[2] University of Milan, Department of Food, Environmental and Nutritional Sciences (DEFENS), Via Celoria 2, 20133 Milan, Italy

[3] RWTH Aachen University, ABBt – Aachen Biology and Biotechnology, 52074 Aachen, Germany

*** Correspondence:** Jun.-Prof. Dr. Dörte Rother, do.rother@fz-juelich.de

1 Inhalt

S1 SDS-Gel from applied enzymes 2
S2 Mass spectrometry analysis of Citrate-1,5-^{13}C flux 2
S2.1 Mass spectrometric chromatogram of detected metabolites 2
S2.1.1 Data of reactions with LbADH CCE 3
S2.1.2 Data of reactions with KRED1-Pglu 4
S2.2 Negative control of time resolved total ion (TIC) chromatograms of enzymatic reaction with LbADH 5
S2.3 Time resolved total ion (TIC) chromatogram of enzymatic reaction with KRED1-*Pglu* 6
S2.4 Time resolved extracted ion chromatogram (XIC) of enzymatic reaction with KRED1-*Pglu* 7
S3 Estimations of different metabolites 8
S3.1 Estimation of total NADP$^+$ amounts 8
S3.2 Estimation of acetyl-CoA concentrations 9
S4 ^{13}C transition of [1,5-^{13}C] citrate in comparison to [2,4-^{13}C]citrate 10

S1 SDS-Gel from applied enzymes

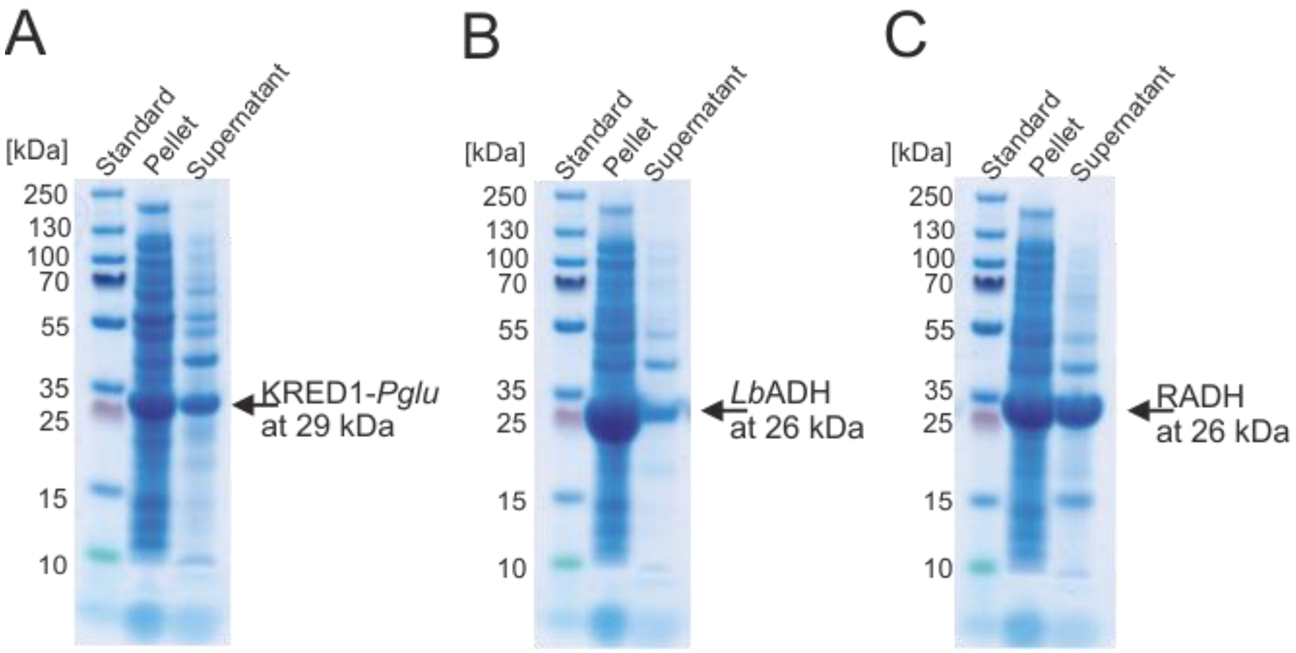

Figure 1: Soluble and insoluble protein fractions of the crude cell extracts, that overexpress KRED1-*Pglu* (A), *Lb*ADH (B), or RADH (C); The whole cell preparation contained all proteins of the insoluble pellet fraction and soluble supernatant fraction; the crude cell extract contained supernatant and not enzymes present in the pellet.

S2 Mass spectrometry analysis of Citrate-1,5-^{13}C flux

S2.1 Mass spectrometric chromatogram of detected metabolites

S2.1.1 Data of reactions with LbADH CCE

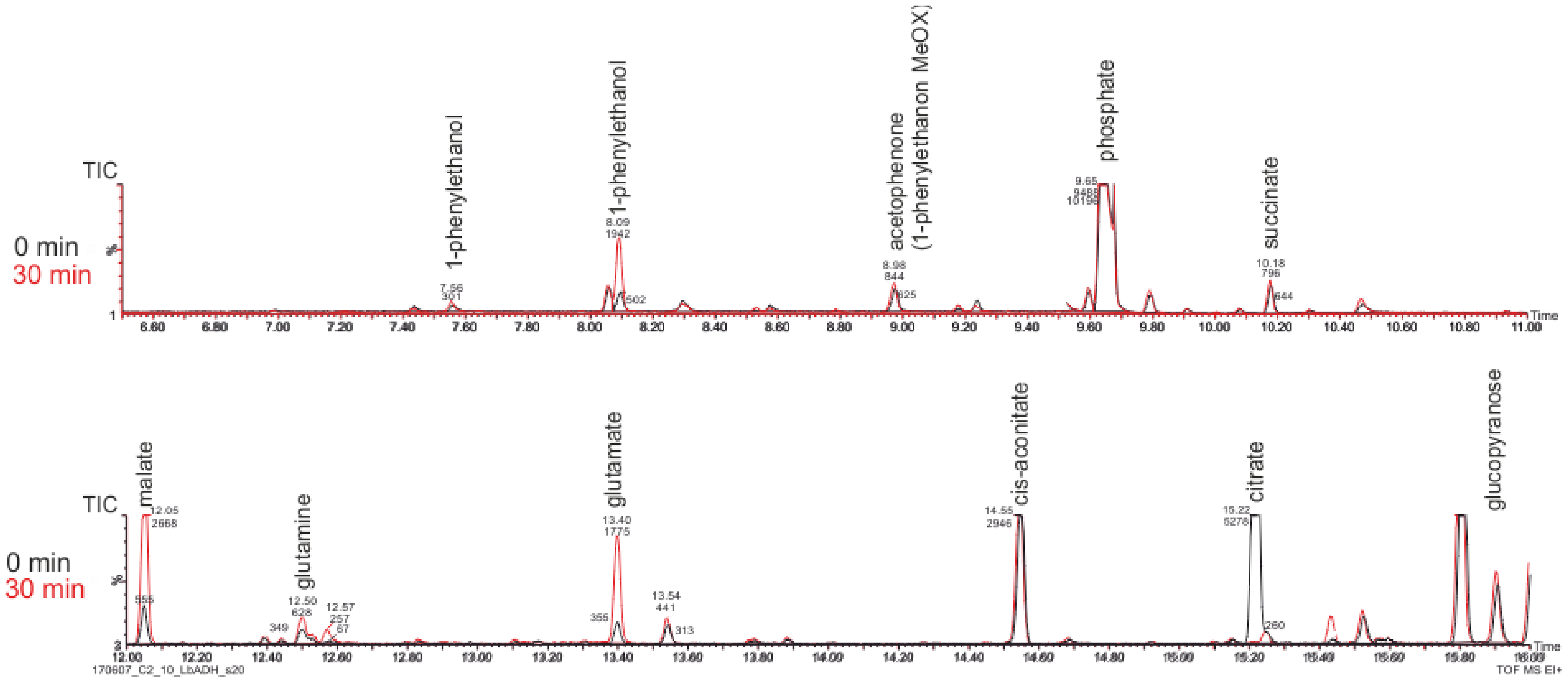

Figure 2: Metabolite profile of citrate-1,5-^{13}C conversion in *Lactobacillus brevis* alcohol dehydrogenase with *Escherichia coli* crude cell extract: Crude cell extract of *Lactobacillus brevis* alcohol dehydrogenase (*Lb*ADH) was applied to convert acetophenone to 1-phenylethanol. Since the reaction requires NADPH as co-factor, citrate was applied to regenerate it. Also marked in the chromatogram is cis-aconitate, which is an intermediate in the aconitase reaction of citrate to isocitrate. Glucopyranose is a cellular substance, which has no impact on the presented hypothesis. Reaction conditions: 20 mg mL^{-1} *Lb*ADH crude cell extract, 5 mM acetophenone, 10 mM citrate, 40 mM potassium-phosphate buffer (concentration was lowered due to MS analytics); 30 °C, 1000 rpm

S2.1.2 Data of reactions with KRED1-Pglu

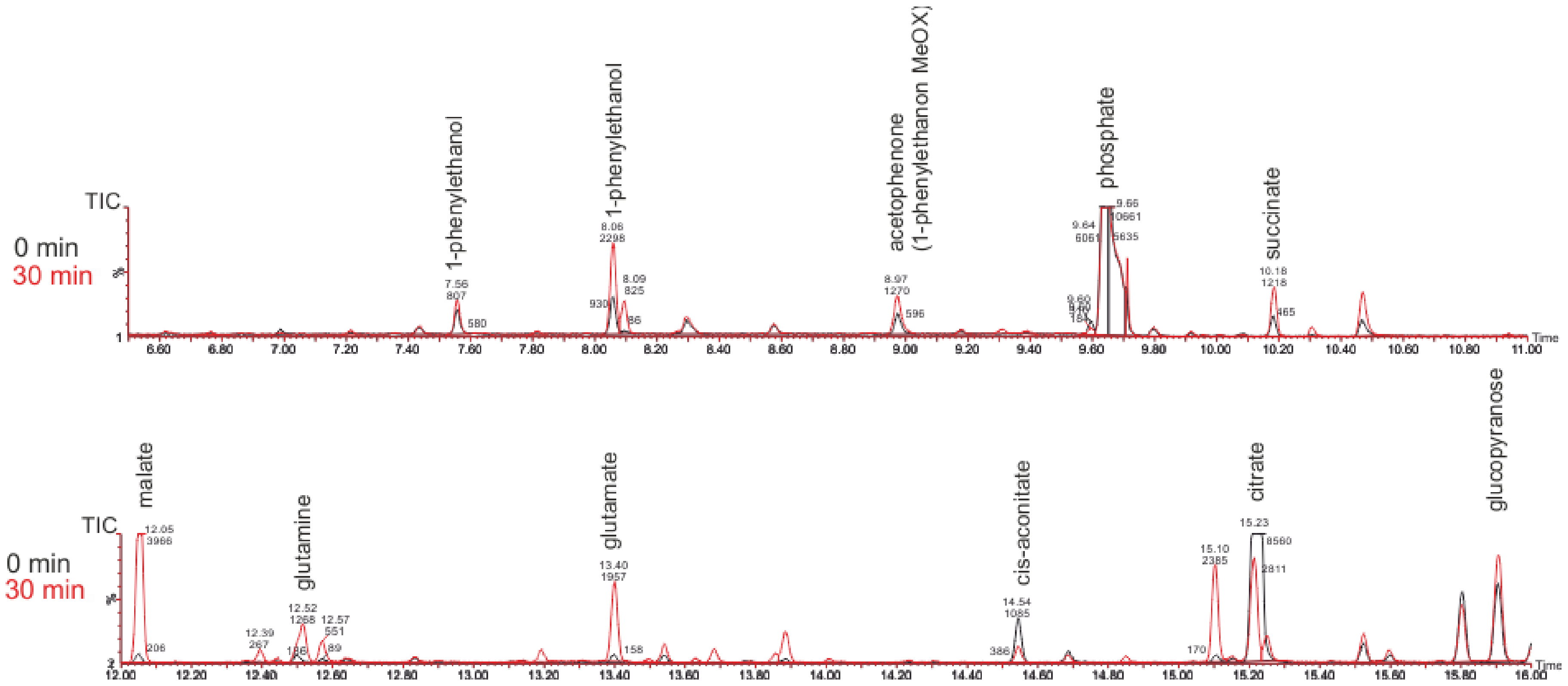

Figure 3: Metablite profile analysis of citrate-1,5-^{13}C conversion in *Ogataea glucozyma* CBS 5766 ketoreductase 1 with *Escherichia coli* crude cell extract: Crude cell extract of *Ogataea glucozyma* CBS 5766 ketoreductase 1 (KRED1-*Pglu*) was applied to convert acetophenone to 1-phenylethanol. Since the reaction requires NADPH as co-factor, citrate was applied to regenerate it. Also marked in the chromatogram is cis-aconitate, which is an intermediate in the aconitase reaction of citrate to isocitrate. Glucopyranose is a cellular substance, which has no impact on the presented hypothesis. Reaction conditions: 20 mg mL^{-1} *Pg*KRED1 crude cell extract, 5 mM acetophenone, 10 mM citrate, 40 mM potassium-phosphate buffer (concentration was lowered due to MS analytics); 30 °C, 1000 rpm

S2.2 Negative control of time resolved total ion (TIC) chromatograms of enzymatic reaction with LbADH

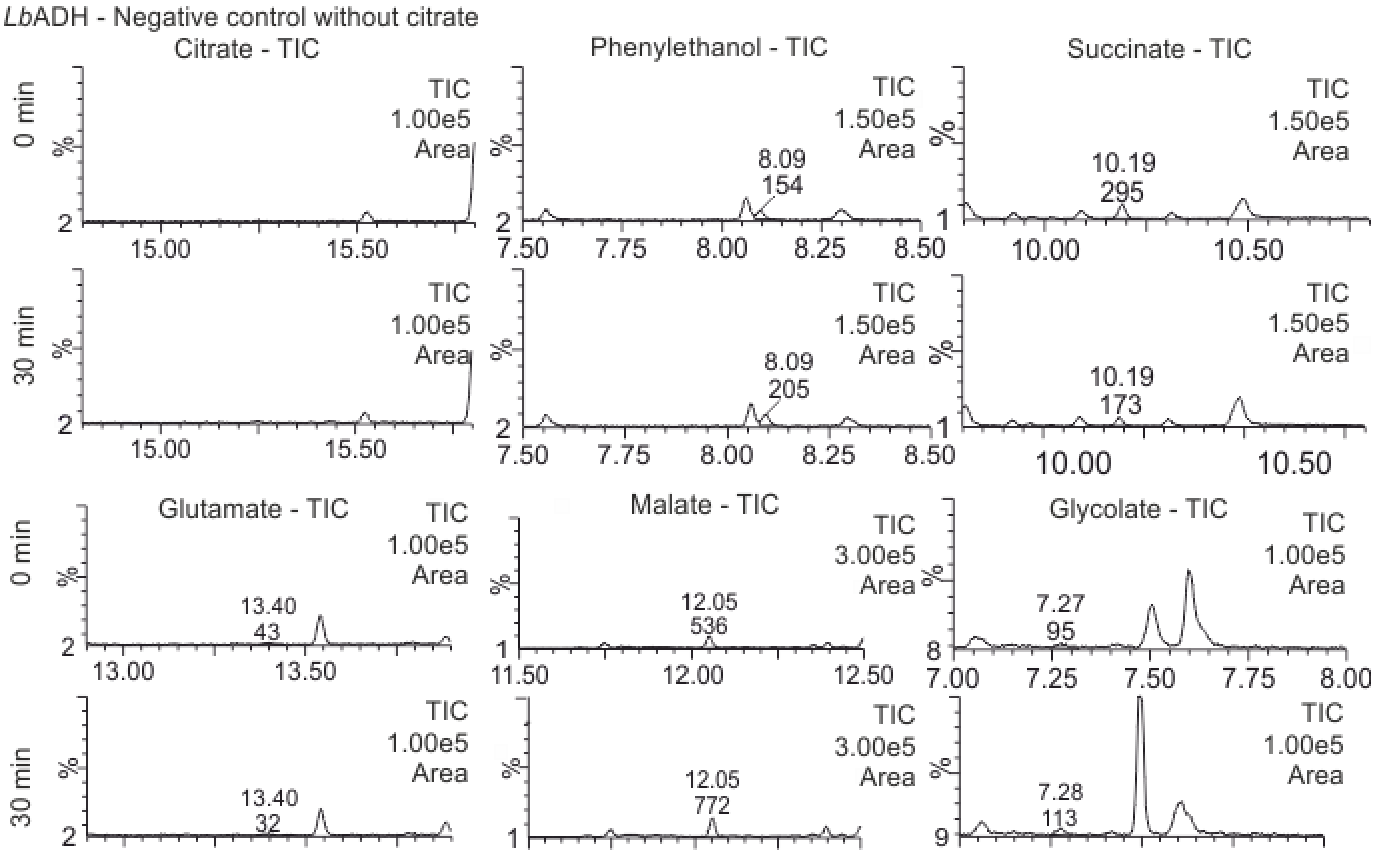

Figure 4. Negative control of total ion chromatograms (TIC) of selected TCA cycle intermediates. Relative concentration changes during the isotopic labeling experiment with [1,5-^{13}C]citrate in *Lactobacillus brevis* alcohol dehydrogenase (ADH) crude cell extract are shown. Reaction conditions: 20 mg mL^{-1} *Lb*ADH CCE, 5 mM acetophenone in 40 mM KP_i buffer pH 6.5; 30 °C, 1000 rpm

S2.3 Time resolved total ion (TIC) chromatogram of enzymatic reaction with KRED1-*Pglu*

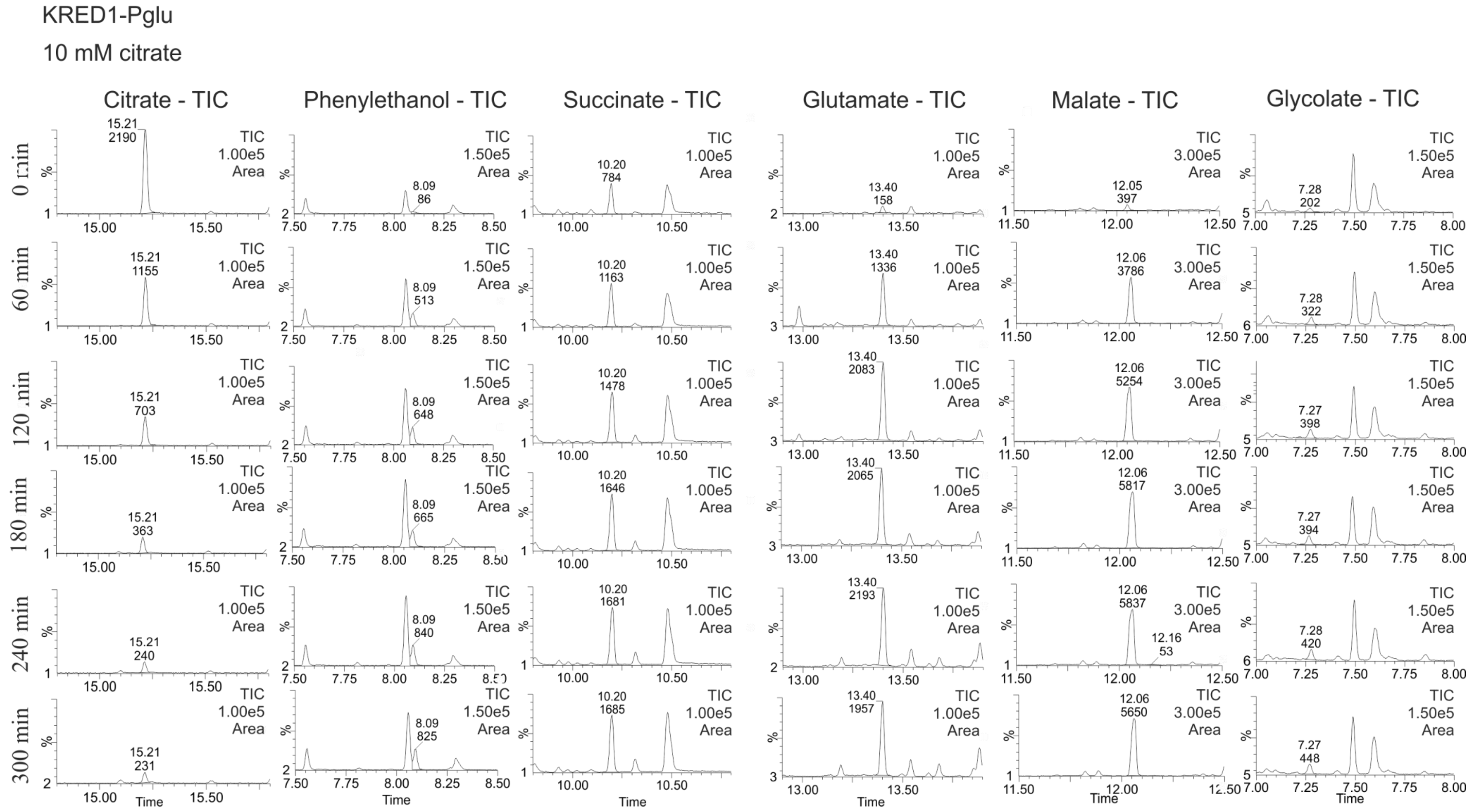

Figure 5. Total ion chromatograms of selected [1,5-^{13}C]citrate metabolites in *Ogataea glucozyma* CBS 5766 ketoreductase 1 (KRED1-*Pglu*) crude cell extract: displayed is the total ion chromatogram (TIC) for extracted ion chromatogram (XIC), which is particular for ^{13}C-labelled citrate, 1-phenylethanol, succinate, glutamate, malate and glycolate. Hence, an increase or dissipation of ^{13}C-labelled

compounds over time is observable. Reaction conditions: 20 mg mL^{-1} KRED1-*Pglu*, 5 mM acetophenone, 10 mM citrate in 40 mM KP_i buffer pH 6.5; 30 °C, 1000 rpm; Note: citrate and isocitrate behaved identical in the analysis and are thus not distinguished in the plot.

S2.4 Time resolved extracted ion chromatogram (XIC) of enzymatic reaction with KRED1-*Pglu*

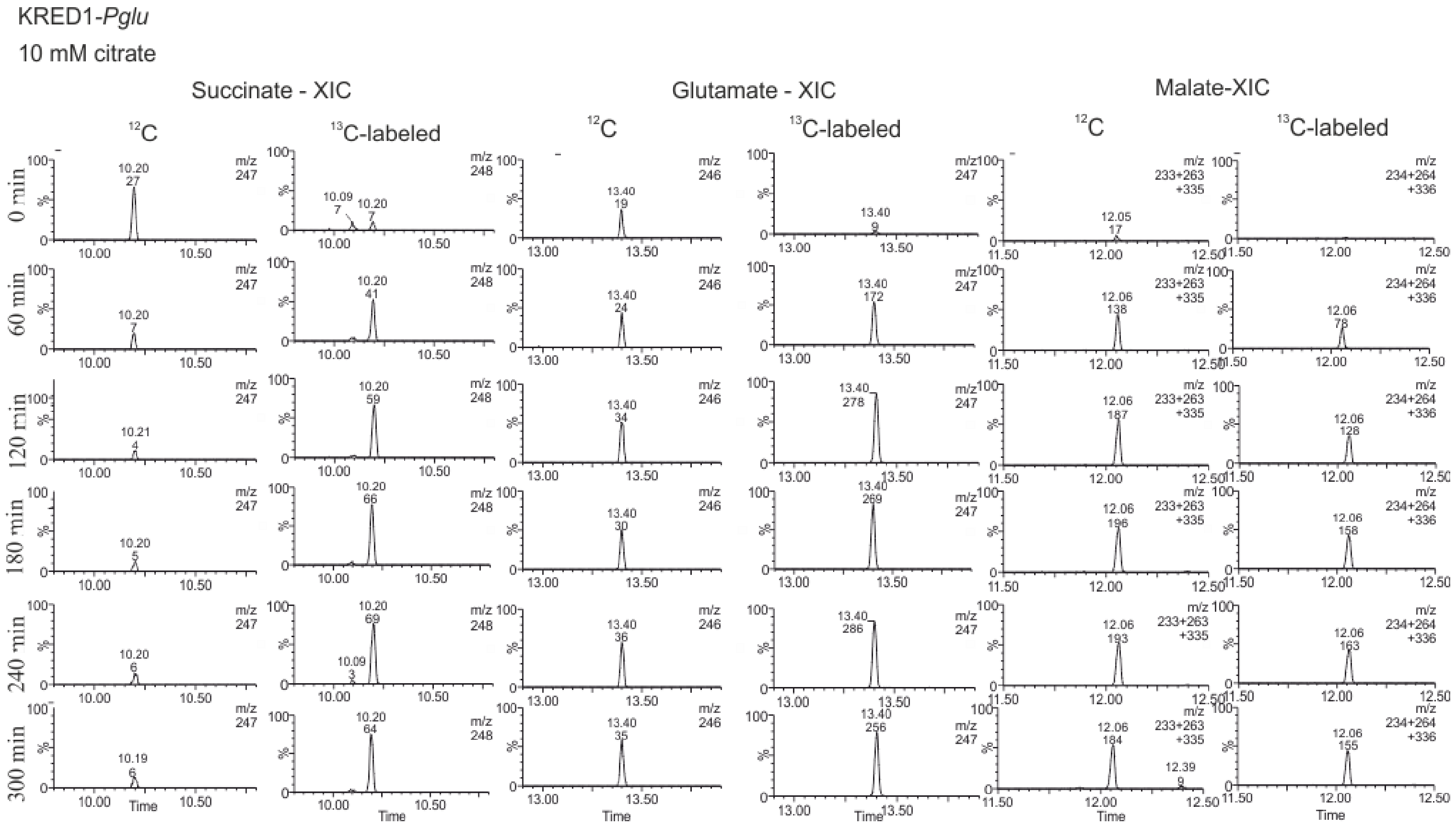

Figure 6. Extracted ion chromatograms (XIC) of selected [1,5-^{13}C]citrate metabolites in *Ogataea glucozyma* CBS 5766 ketoreductase 1 (KRED1-*Pglu*) crude cell extract: The accumulation and dissipation of ^{12}C and ^{13}C labeled succinate, glutamate, and malate is displayed in detail. KRED1-*Pglu* has longer reaction times than *Lb*ADH. Reaction conditions: 20 mg mL^{-1} KRED1-*Pglu*, 5 mM acetophenone, 10 mM citrate in 40 mM KP_i buffer pH 6.5; 30 °C, 1000 rpm;

S3 Estimations of different metabolites

S3.1 Estimation of total $NADP^+$ amounts

The intracellular NADP/NADPH concentration is according to Bennett *et al*. 0.12 mM and according to Chemler 0.33 mM (Bennett et al., 2009; Chemler et al., 2010). According to Nöh *et al.* the intracellular volume of *E. coli* accounts for 2.78 mL per 1 g dry cell weight (Nöh et al., 2007). In the herein applied conditions 20 mg crude cell extract from 20 mg lyophilized whole cells are applied. Hence the total intracellular volume is 0.0556 mL. The total molecule amount can be calculated by first calculating the amount of substance (n) and then calculating the number of constituent molecules (N).

	Bennett *et al.*	Cemler *et al.*
$n = c * V$	$0.056\, mL * 0.12\, mM = 6.67 * 10^{-3} mol$	$0.056\, mL * 0.33\, mM = 1.83 * 10^{-2} mol$
$N = n * N_A$	$6.67 * 10^{-3} mol * 6.022 * 10^{23}\, mol^{-1} = 4.01 * 10^{21}$	$1.83 * 10^{-2} mol * 6.022 * 10^{23}\, mol^{-1} = 1{,}10 * 10^{22}$

N_A is the Avogadro number = $6.022*10^{23}$ particles

Hence the reaction setup contains approximately a **total number of NADP/NADPH molecules in the magnitude of 10^{21} to 10^{22}.**

This number can be compared against an estimated number of total heterologous protein. One single *E. coli* cell contains a total of 3600 proteins. Assuming a worst case scenario and saying that all of this would represent NADPH-dependent proteins (which is not the case), would mean 3600 protein per one cell. One cell weighs on average in dry state 300 fg (Milo, 2018; Neidhardt et al., 1990). Hence, 20 mg of lyophilized whole cells consists of 10^{10} cells. This means in a worst case scenario $2.4*10^{14}$ NADPH-dependent molecules.

Calculating method:

1 fg = 10^{-15}g → if 1 cell = 300 fg then 20 mg = $6.7*10^{10}$ cells

→ If 1 cell = 3600 proteins then $6.7*10^{10}$ cells contain $2.4*10^{14}$

A comparison between the estimated magnitude of total NADP/H molecules and total possible NADPH-dependent enzymes, shows that NADP is by far in excess → $10^{21} >> 10^{14}$.

S3.2 Estimation of acetyl-CoA concentrations

For acetyl-CoA an intracellular concentration of $6.1*10^{-4}$ mol L^{-1} (Bennett et al., 2009). According to Nöh *et al.* the intracellular volume of *E. coli* accounts for 2.78 mL per 1 g dry cell weight (Nöh et al., 2007). In the herein applied conditions 20 mg crude cell extract from 20 mg lyophilized whole cells are applied in a volume of 1 mL. Hence, first the total cell volume in the setup is calculated, then the concentration of acetyl-CoA in this volume is calculated. From this the concentration in the total reaction volume is calculated.

The total cell volume is $2.78\frac{ml}{g} * 0.02\ g = \ 0.0556\ mL$ = V1

C1=$6.1*10^{-4}$ mol L^{-1}

V2= $1*10^{-3}$ L

$$c1 * V1 = c2 * V2 \rightarrow c2 = \frac{6.1*10^{-4} mol}{L} * 5.56 * 10^{-5} L: 0.001L$$

C2 = c(acetyl-CoA) =34 µmol L^{-1} = 0.034 mM

In the applied cell amount acetyl-CoA has a concentration of $6.1*10^{-4}$ mol L^{-1}. Hence, its concentration in the whole setup refers to 34 µmol L^{-1}.

S4 ^{13}C transition of [1,5-^{13}C] citrate in comparison to [2,4-^{13}C]citrate

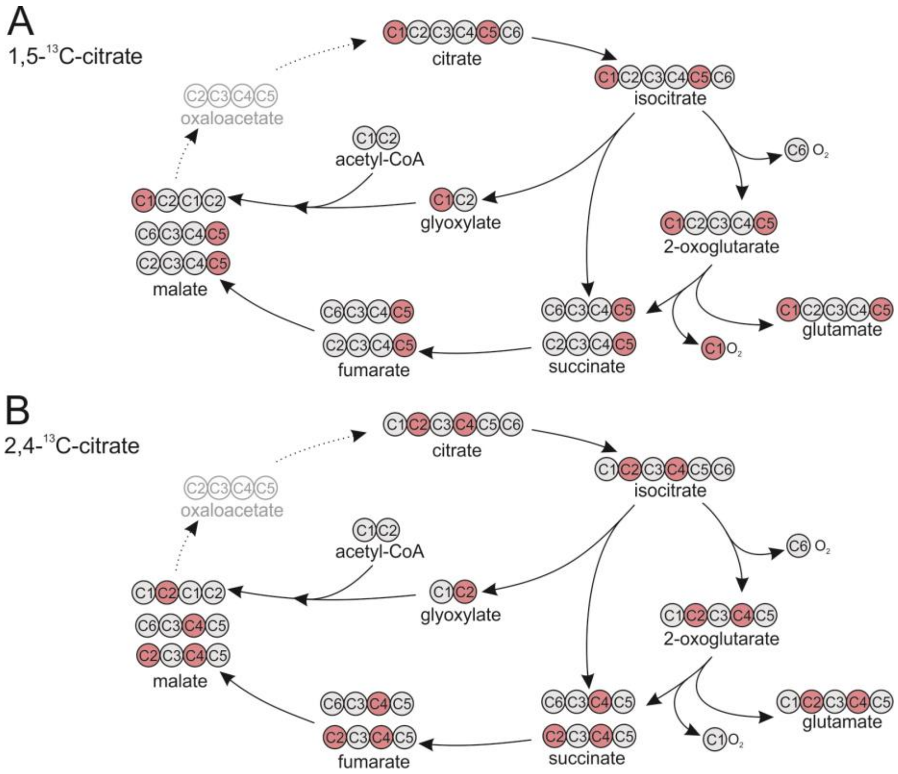

Figure 7. ^{13}C transition in metabolites when [1,5-^{13}C]citrate is supplemented (A) and [2,4-^{13}C]citrate (B). A ^{13}C labelling of citrate in position 2 and 4 would enable a ratio calculation between the different pathways citrate takes in the performed CCE setup.

References

Bennett, B.D., Kimball, E.H., Gao, M., Osterhout, R., Van Dien, S.J., Rabinowitz, J.D., 2009. Absolute metabolite concentrations and implied enzyme active site occupancy in *Escherichia coli*. Nature Chemical Biology 5, 593–599. https://doi.org/10.1038/nchembio.186

Chemler, J.A., Fowler, Z.L., McHugh, K.P., Koffas, M.A.G., 2010. Improving NADPH availability for natural product biosynthesis in *Escherichia coli* by metabolic engineering. Metabolic Engineering 12, 96–104. https://doi.org/10.1016/j.ymben.2009.07.003

Milo, R., 2018. Overall macromolecular composition of *E. coli* cell [WWW Document]. URL http://bionumbers.hms.harvard.edu/bionumber.aspx?id=111490&ver=7&trm=E. coli weight&org=

Neidhardt, F.C., Tummler, K., Milo, R., 1990. Physiology of the bacterial cell. Sinauer.

Nöh, K., Grönke, K., Luo, B., Takors, R., Oldiges, M., Wiechert, W., 2007. Metabolic flux analysis at ultra short time scale: Isotopically non-stationary ^{13}C labeling experiments. Journal Of Biotechnology 129, 249–267. https://doi.org/10.1016/j.jbiotec.2006.11.015
